# Prognostic Value and Targeted Inhibition of Survivin Expression in Esophageal Adenocarcinoma and Cancer-Adjacent Squamous Epithelium

**DOI:** 10.1371/journal.pone.0078343

**Published:** 2013-11-04

**Authors:** Usha Malhotra, Ali H. Zaidi, Juliann E. Kosovec, Pashtoon M. Kasi, Yoshihiro Komatsu, Christina L. Rotoloni, Jon M. Davison, Clint R, Irvin, Toshitaka Hoppo, Katie S. Nason, Lori A. Kelly, Michael K. Gibson, Blair A. Jobe

**Affiliations:** 1 Department of Medicine, Roswell Park Cancer Institute, Buffalo, New York, United States of America; 2 Department of Medicine, University of Pittsburgh, Pittsburgh, Pennsylvania, United States of America; 3 Allegheny-Singer Research Institute, Allegheny Health Network, Pittsburgh, Pennsylvania, United States of America; 4 Department of Pathology, University of Pittsburgh, Pittsburgh, Pennsylvania, United States of America; 5 Department of Surgery, Allegheny Health Network, Pittsburgh, Pennsylvania, United States of America; 6 Department of Cardiothoracic Surgery, University of Pittsburgh, Pittsburgh, Pennsylvania, United States of America; University of Pittsburgh, United States of America

## Abstract

**Background:**

Survivin is an inhibitor of apoptosis and its over expression is associated with poor prognosis in several malignancies. While several studies have analyzed survivin expression in esophageal squamous cell carcinoma, few have focused on esophageal adenocarcinoma (EAC) and/or cancer-adjacent squamous epithelium (CASE). The purpose of this study was 1) to determine the degree of survivin up regulation in samples of EAC and CASE, 2) to evaluate if survivin expression in EAC and CASE correlates with recurrence and/or death, and 3) to examine the effect of survivin inhibition on apoptosis in EAC cells.

**Methods:**

Fresh frozen samples of EAC and CASE from the same patient were used for qRT-PCR and Western blot analysis, and formalin-fixed, paraffin-embedded tissue was used for immunohistochemistry. EAC cell lines, OE19 and OE33, were transfected with small interfering RNAs (siRNAs) to knockdown survivin expression. This was confirmed by qRT-PCR for survivin expression and Western blot analysis of cleaved PARP, cleaved caspase 3 and survivin. Survivin expression data was correlated with clinical outcome.

**Results:**

Survivin expression was significantly higher in EAC tumor samples compared to the CASE from the same patient. Patients with high expression of survivin in EAC tumor had an increased risk of death. Survivin expression was also noted in CASE and correlated with increased risk of distant recurrence. Cell line evaluation demonstrated that inhibition of survivin resulted in an increase in apoptosis.

**Conclusion:**

Higher expression of survivin in tumor tissue was associated with increased risk of death; while survivin expression in CASE was a superior predictor of recurrence. Inhibition of survivin in EAC cell lines further showed increased apoptosis, supporting the potential benefits of therapeutic strategies targeted to this marker.

## Introduction

Esophageal cancer is currently the eighth most common cancer worldwide; with esophageal adenocarcinoma (EAC) accounting for 50% of esophageal cancer cases [1]–[5]. The five-year survival for esophageal cancer is less than 20% and its incidence has increased by almost three fold in the western hemisphere in the past 20 years [6], [7]. Most EAC patients are diagnosed with advanced stage disease and have poor long-term survival rates with the currently employed chemotherapeutic agents [5], [8], [9]. The efficacy of current regimens has reached a plateau and further intensification of cytotoxic agents or radiation dose escalation has been shown to be associated with significant adverse side effects. Consequently, the need for the development of effective targeted therapies aimed at treating specific mechanisms of carcinogenesis are required in order to improve survival [2]–[4], [10], [11].

**Figure 1:**
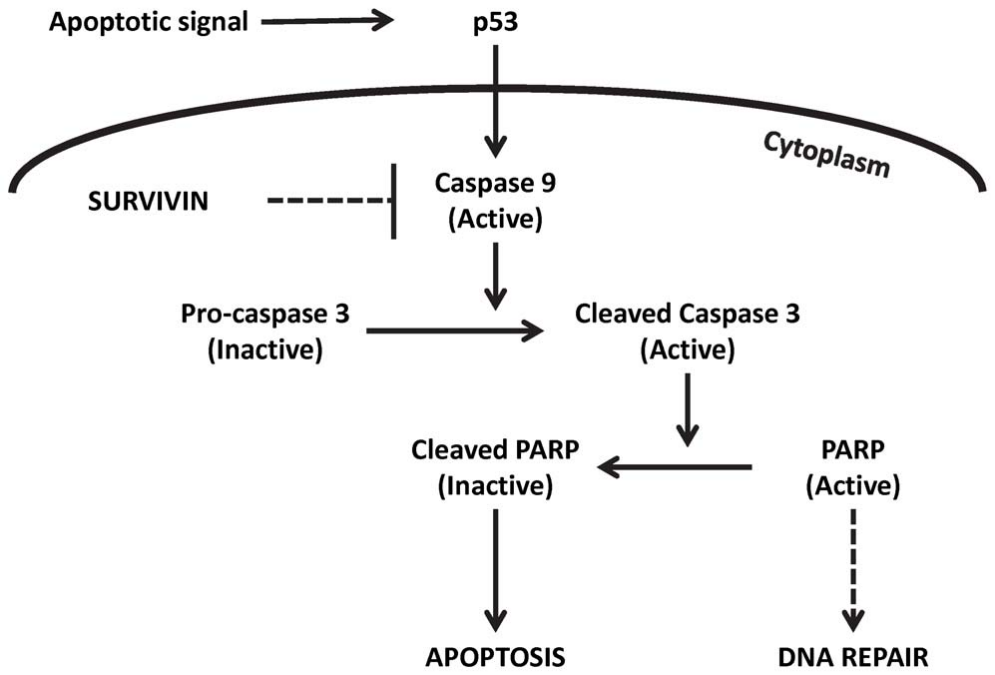
Survivin inhibition pathway. Survivin binds to and inhibits caspase 9, caspase 9 is unable to cleave caspase 3, caspase 3 is unable to cleave PARP, PARP promotes DNA repair and does not induce apoptosis.

Survivin, also known as Baculoviral Inhibitor of apoptosis Repeat-Containing 5 or BIRC5, is an inhibitor of apoptosis or programmed cell death [10]–[13]. The mechanism of action through the intrinsic pathway is as follows: survivin binds to and inhibits caspase 9, causing deactivation of the apoptotic pathway; procaspase 3 is not cleaved and thus does not cleave PARP (Poly ADP-ribose polymerase); as a result, PARP remains active and continues with DNA repair, resulting in the inhibition of apoptosis (**Figure 1**) [8], [10], [11], [13], [14]. Ordinarily survivin is only found during embryonic and fetal development as a means to regulate proper cell division and growth and is undetectable in most terminally differentiated normal tissues [15]. Some normal adult tissues with persistent survivin expression include hematopoietic stem cells [16], thymocytes [17], melanocytes [18], gastric mucosa [19] and colonic epithelium [19]. Studies have reported increased expression of survivin in a number of cancers including breast, lung, melanoma, leukemia, lymphoma, colon, pancreas, and etc. [15]. Evidence also supports over expression of survivin in esophageal squamous cell carcinoma and its association with a poor prognosis [3], [4], [8], [10], [11], [20]. Because survivin is not expressed in the majority of healthy tissues, it represents an ideal target for the development of novel cancer agents [3], [4], [8], [10], [11], [13], [20], [21]. In fact agents targeting survivin are currently in phase I and phase II clinical trials in patients with advanced cancer where methods of inhibition include antisense oligonucleotides (ASOs), transcriptional repressors, and immunotherapy [10], [14], [22]. Some of these agents have demonstrated promising initial results, however none have improved overall survival [3], [4], [7], [10], [11], [14], [22]–[24].

Although there have been many reports analyzing the role of survivin in esophageal squamous cell carcinoma (SCC), very few studies have addressed its role in esophageal adenocarcinoma (EAC) [3], [4], [7]–[9], [21], [25], [26]. In addition expression of this anti-apoptotic gene in the cancer-adjacent squamous epithelium (CASE) has not been studied. The purpose of our study, therefore, was 1) to determine the degree of survivin up regulation in samples of EAC patients and CASE, 2) to evaluate recurrence and survival with survivin expression in EAC and CASE tissue, and 3) to examine the effect of survivin inhibition on apoptosis in EAC cell lines.

## Materials and Methods

### Ethics Statement

The study was performed after obtaining approval from University of Pittsburgh Institutional Review Board. Samples were taken from the UPMC Cancer Center - Esophageal Cancer Risk Registry, University of Pittsburgh IRB Study Number 98–122 with URL: http://www.upmccancercenter.com/trials/trialDisplay.cfm?id=2277&type=D. As a part of the study, all patient samples were obtained with full written consent.

### Cell Lines

The EAC cell lines OE19 (JROECL19) and OE33 (JROECL33) were obtained through Sigma-Alderich (St. Louis, MO). They were both maintained in Roswell Park Memorial Institute (RPMI) 1640 cell growth media (Life Technologies, Carlsbad, CA, 21870076), supplemented with 10% Fetal bovine serum (FBS) (Life Technologies, Carlsbad, CA, 26140079), 1% L-Glutamine (Life Technologies, Carlsbad, CA, 25030081), 1% Penicillin-Streptomycin (Life Technologies, Carlsbad, CA, 15070063). All cells were stored in 25 cm^2^ flasks and incubated at 37°C with 5% CO_2_ humidified air. Trypsin-EDTA (Life Technologies, Carlsbad, CA, 25200072) was used to harvest the cells from their flask and prepare a single cell suspension for Western blot analysis and Reverse Transcription – Polymerase Chain Reaction (RT-PCR).

### siRNA Transfection

OE33 and OE19 were seeded in a 6-well plate at a density of 4×10^5^ 24 hours before transfection. Cells were either transfected with small interfering RNAs (siRNAs) or untreated. The siRNA categories utilized included survivin specific siRNA, or negative control (scramble) (Dharmacon, Lafayette, CO, D-001810-10-05). Each siRNA solution was diluted from the 100 µM stock to 5 µM in RNase-free H_2_O, then diluted to 0.25 µM in Opti-MEM (Life Technologies, Carlsbad, CA, 31985062). Simultaneously, 2 µL of transfection reagent 4 (Dharmacon, Lafayette, CO, T-2004) per reaction was diluted with 98 µL of Opti-MEM. The solutions were separately incubated for 5 minutes at room temperature, mixed together, and incubated for another 20 minutes at room temperature. The final siRNA solution was diluted to 1 mL per reaction with Opti-MEM for a final concentration of 25 ηM. The cells were incubated at 37°C in 5% CO_2_ humidified air, with a RPMI media change after 24 hours. Whole cell lysate was collected at 48 hours for RNA analysis or 72 hours for protein analysis.

### Patients and Tissue Preparation

The study was performed after obtaining approval from University of Pittsburgh Institutional Review Board. Patients who underwent esophagectomy for localized esophageal adenocarcinoma from beginning of 2008 to end of 2010 were included in the study. Forty-seven samples were screened and after excluding squamous cell carcinoma, adenosquamous cancer, and tissue blocks with less than 70% tumor, a total of 37 patient samples were analyzed for this study. Clinical data including age, sex, ethnicity, clinical stage, vital status, smoking history, overall survival, and time to recurrence was obtained from the Department of Cardiothoracic Surgery clinical database. To ensure patient confidentiality, an honest broker system was utilized to provide de-identified clinical dataset linked to tissue samples [27]. Fresh frozen tissue embedded in OCT was used for quantitative reverse transcription-polymerase chain reaction (qRT-PCR) and Western blot analysis. Formalin-fixed, paraffin-embedded (FFPE) tissue was used for immunohistochemistry.

### Western Blot Analysis

Protein was collected from each cell line treatment group by adding lysis buffer (RIPA buffer containing 0.1% protease inhibitor cocktail mix, 0.1% Halt phosphatase inhibitor cocktail mix and 1% PMSF) and using a cell lifter. The solution was rotated for one hour at 4°C to fully lyse the cells, then spun at 12,000 g at 4°C for 20 minutes to separate the protein. The supernatant containing the protein was collected and quantified using BCA Pierce Assay (Thermofisher, Rockford, IL, 23227). Thirty-five µg of protein from each sample was denatured and resolved by 4–20% SDS-PAGE gradient gel (Bio-Rad, Hercules, CA, 161–1159), then electroblotted to a Immobilon-P PVDF nitrocellulose membrane (Millipore Corporation, Billerica MA, IPVH08100). The membrane was blocked in 5% non-fat dried milk in TBS-T at room temperature for one hour. The antibody of interest was incubated with the membrane at 4°C overnight, followed by 1.5 hour incubation at room temperature in the corresponding horseradish peroxidase-conjugated secondary antibody (Cell Signal, Boston MA, 7074 or 7076, 1∶3,000) Survivin antibody (Santa Cruz Biotechnology, Dallas, Tx, sc-47750) was used at 1∶200, caspase-3 (Cell Signal, Boston, MA, 9665) at 1∶1,000, cleaved-PARP (Cell Signal, Boston, MA, 9546) at 1∶2,000 and β-actin (Sigma-Aldrich, St. Louis, MO, A1978) at 1∶30,000 was used as a loading control. Signals were developed using a chemiluminescence reagent (Fisher Scientific, Pittsburgh, PA, 509049324).

### Quantitative Reverse Transcription – Polymerase Chain Reaction (qRT-PCR)

Total RNA was purified from cell lines using the RNeasy Micro Kit (Qiagen, Valencia, CA, 74004). Briefly, pellets containing 1×10^6^ cells were vortexed in RLT lysis buffer and RNA was extracted following manufacturer's protocol using an elution volume of 14 ul. Using an ND-2000 spectrophotometer (Nanodrop Technologies, Inc, Wilmington, DE), the quality and quantity of RNA was assessed by OD_260/280_. Complementary DNA (cDNA) was reverse transcribed from 3 ug total RNA using RNA to cDNA ecodry premix in a total volume of 20 ul (Clontech, Mountain View, CA, 639547) according to the manufacturer's protocol. The reaction conditions were 42°C for 60 minutes, followed by 70°C for 10 minutes using ABI Prism 7900HT PCR thermocycler (Applied Biosystems, Carlsbad, CA). PCR was performed in a total volume of 20 ul using 600 ng cDNA, 1× Taqman PCR universal mastermix (Invitrogen, Grand Island, NY, 4304437), and 1× Survivin or GAPDH primer (Applied Biosystems, Carlsbad, CA hs03043576_m1 and hs99999905_m1) for each reaction. The cycling parameters were: one cycle of 95°C for ten minutes, followed by 45 cycles of 95°C for 15 seconds, and 60°C for one minute on a StepOne Plus system (Applied Biosystems, Carlsbad, CA). All reactions were run in technical duplicates.

Extraction of total RNA from fresh frozen tissue was done using the Qiagen RNeasy Mini kit (Qiagen, Valencia, CA 74104). Briefly, 10 µm OCT sections were cut into RLT buffer, lysed using a 20 gauge blunt end needle and RNA was extracted following manufacturer's instructions using an elution volume of 50 ul. qRT-PCR was performed with the One-step qRT-PCR Kit (Life Technologies, Carlsbad, CA 4310299) according to manufacturer's instructions in a total volume of 20 ul using 2 ul of tissue lysate and 1× Survivin or 1× GAPDH primer (Life Technologies, Carlsbad, CA hs03043576_m1 and hs99999905_m1) for each reaction. The cycle conditions used were: 1 cycle of 50°C for 30 minutes, 95°C for 2 minutes, and 50 cycles of 95°C for 15 seconds, 60°C for 45 seconds using ABI Prism 7700 Sequence Detection System (Applied Biosystems, Carlsbad, CA). All reactions were run in technical triplicates. Amplification plots for both sample categories, cell lines and fresh frozen tissues were examined with StepOne software, provided with the StepOne Plus system, to determine the cycle threshold (CT). GAPDH was used for normalization of expression data. The 2^−ΔCT^ method was used to determine survivin fold expression in tumor tissue and CASE.

### Immunohistochemistry

FFPE sections were immunostained to show survivin expression in EAC tumor and paired squamous epithelial samples. Reactive human tonsil and non-immune serum were run in parallel as positive and negative controls, respectively. Tissue samples were cut at 4 µm onto charged slides and deparaffinized. The slides were immersed in 0.01% Triton X-100 for ten minutes at room temperature, rinsed in tap H_2_O, then submerged in 10 mM citrate buffer, pH 6.0 at 95°C for 20 minutes for antigen retrieval. Blocking occurred by 3% H_2_O_2_ followed by a tap water wash. After three washes of 1× Tris-buffered saline with 0.001% Tween20 (TBS-T), slides were further blocked in 2.5% normal horse serum (Vector Laboratories, Burlingame, CA, MP7401). The survivin primary antibody (Santa Cruz Biotechnology, Santa Cruz, CA, sc47750) was applied at 1∶50, overnight at 4°C. Slides were washed three times in TBS-T, then incubated in ImmPress anti-rabbit reagent (Vector Laboratories, Burlingame, CA, MP7401) for 30 minutes to aid in survivin expression detection. Again, the slides were washed three times in TBS-T, and developed using ImmPact DAB (Vector Laboratories, Burlingame, CA, SK4105).

### Statistical Analysis

Statistical analyses were performed using SPSS software (IBM, Armonk, NY, Version 20). A p-value <0.05 was considered statistically significant. Within each tissue sample, mean values of Survivin mRNA expression levels (determined by 2^−ΔCT^ method) were evaluated in both EAC tumor tissue (tumor survivin level) and CASE tissue samples (normal survivin level). Student's t test was used to compare mean tumor and normal survivin levels. Receiver operating characteristic (ROC) analysis was used to determine if survivin levels could be used to define high and low risk groups for recurrence; the high risk/low risk thresholds established in the ROC analyses were used to categorize patients as high risk/low risk for both CASE tissue survivin levels and EAC tissue survivin levels. Separate ROC analyses were conducted predicting recurrence from CASE tissue survivin levels and from EAC tissue survivin levels. In both cases visual inspection of the produced graphical figures were used in conjunction with listed Survivin score cut points with corresponding sensitivity and (1 minus) specificity in order to determine optimum thresholds to define high and low risk groups.

Regarding the ROC procedure for threshold determination, we used the standard method of visually inspecting the curve to determine that point closest to the upper-left corner of the ROC figure, along with inspection of corresponding sensitivity and specificity values along available values. This provided a means of determining which value provided the best balance between sensitivity and specificity. Upon inspection of the ROC figures, this inspection was relatively straightforward for CASE, however was somewhat less straightforward for tumor cell levels. In that case, a value was chosen that emphasized sensitivity at the expense of specificity, as false negatives were judged to be more dangerous than were false positives. This relied more heavily on the calculated sensitivity/specificity values as there was a non-significant AUC (area under curve) and accordingly no visually apparent cut point.

Kaplan-Meier curve was generated to determine survival based on high and low risk recurrence groups. A Mantel-Cox Log Rank Test was conducted in order to compare overall survival between risk groups. A Cox proportional hazards model was used to assess the effectiveness of survivin level within both CASE and EAC tissue samples in predicting mortality.

## Results

### Overexpression of survivin in EAC cell lines, human tumor and CASE tissue

**Figure 2:**
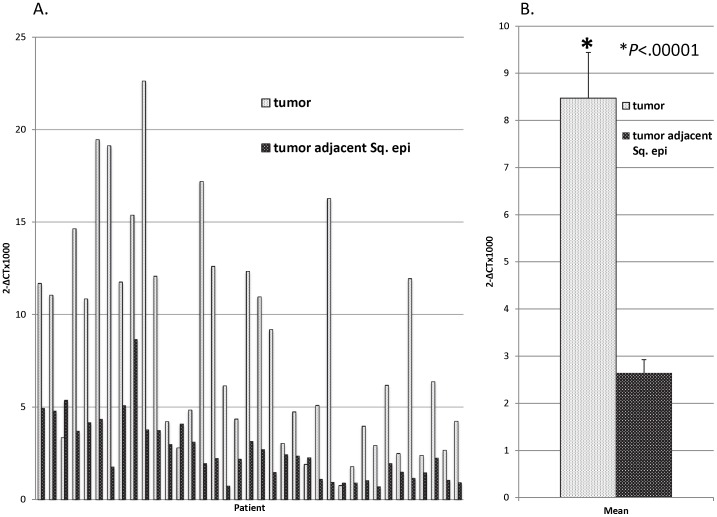
Quantitative real time polymerase chain reaction (qRT-PCR) of patient survivin levels. A: qRT-PCR was performed to compare survivin expression between tumor and adjacent squamous epithelial tissue. B: On average, tumor samples showed 3× greater survivin expression than paired adjacent tissue.

**Figure 3:**
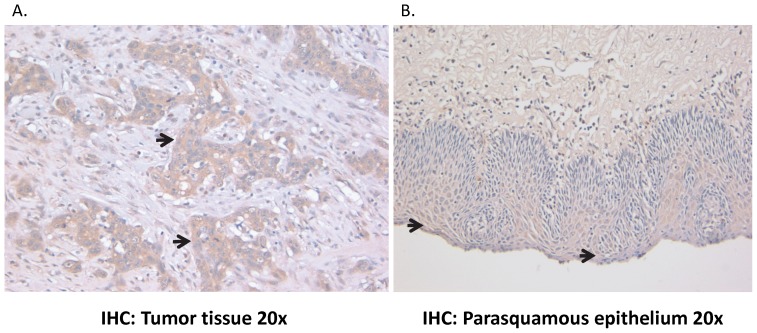
Immunohistochemistry of survivin in human tissue. Survivin expression was evaluated through IHC staining, tumor tissue and adjacent squamous epithelium showed presence of staining indicative of survivin expression. Arrows represent positive staining.

Survivin mRNA expression was significantly higher in tumor samples when compared to CASE tissue samples on qRT-PCR analysis (**Figure 2A**). On average, tumor samples demonstrated levels of survivin expression 3× greater than that of CASE tissue (p<0.00001. **Figure 2B**). Immunohistochemistry (**Figure 3**) and Western Blot analysis (data not shown) also confirmed survivin expression in both EAC tumor as well as CASE.

### Inhibition of survivin in EAC cell lines resulted in increased apoptosis

**Figure 4:**
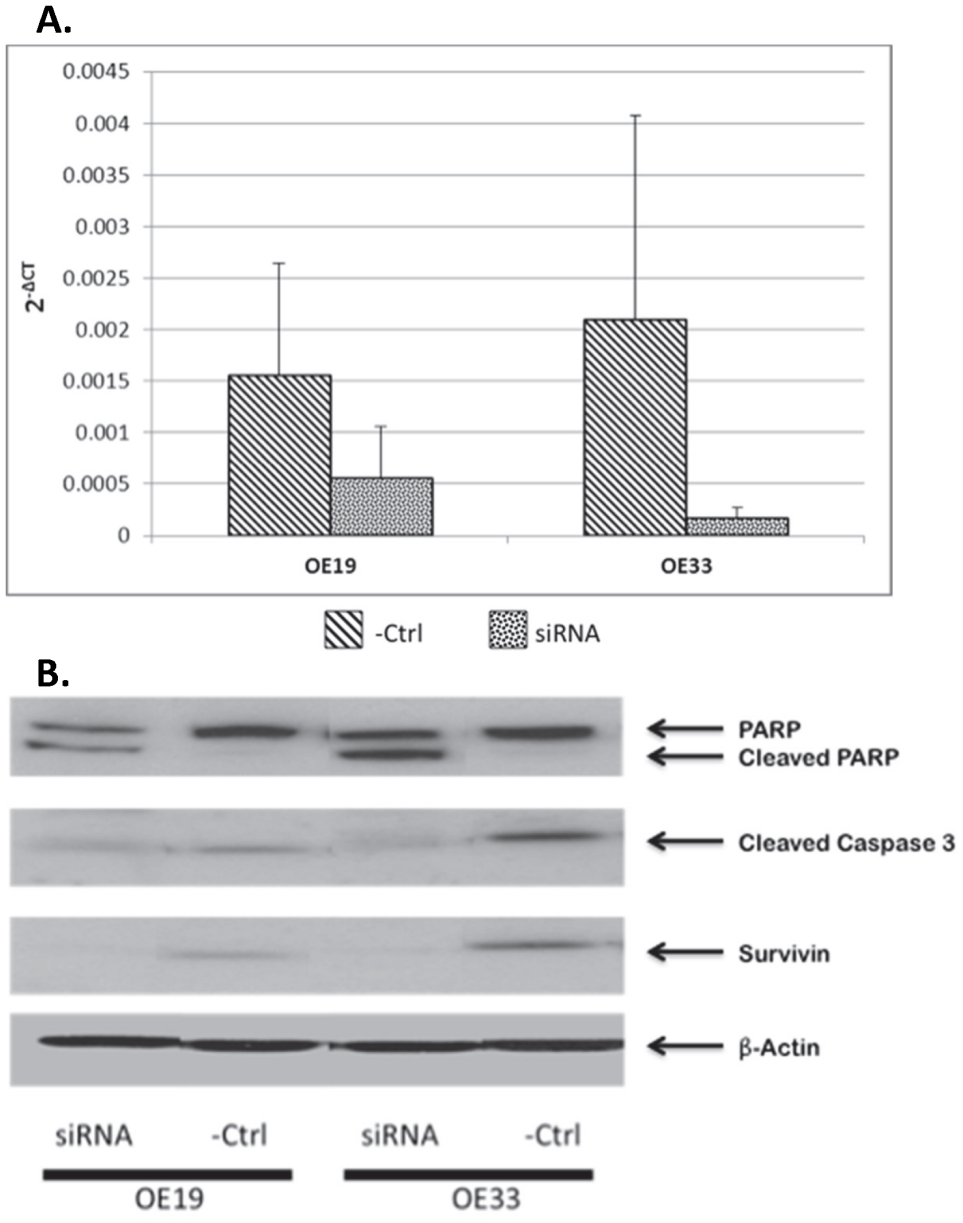
A: Reverse transcriptase polymerase chain reaction of siRNA inhibition of survivin in EAC cell line. OE19 and OE33 EAC cell lines were transfected with siRNA and survivin expression was analyzed between control and transfected cell lines. Both cell lines transfected with siRNA showed clear inhibition and downregulation of survivin expression. **B: Western blot of siRNA inhibition of survivin in EAC cell line.** siRNA was incubated with OE19 and OE33 cell lines to inhibit survivin expression. β-Actin was used as a loading control. Survivin was downregulated, while downstream apoptotic proteins cleaved caspase 3 and cleaved PARP were upregulated. The upregulation of downstream apoptotic proteins indicates apoptosis occurs through incubation with siRNA.

siRNA targeted to survivin resulted in decreased expression of survivin in OE19 and OE33 cell lines compared to controls on qRT-PCR (**Figure 4A**). Similarly, siRNA incubation resulted in downregulation of survivin expression in both cell lines on Western Blot analysis. The decrease in survivin expression resulted in upregulation of the downstream apoptotic protein, cleaved PARP. Cleaved caspase 3 was consumed through the reaction with PARP, resulting in upregulation of cleaved PARP (**Figure 4B**).

### Clinical implications of survivin overexpression

Thirty-seven EAC patients who underwent esophagectomy for localized esophageal adenocarcinoma were included in the study (**Table 1**). The samples comprised thirty males (81%) and seven females (19%) with ages ranging from 44 to 90 years (mean = 62.76, sd = 10.94). Stage distribution of the study cohort based on The American Joint Committee on Cancer (AJCC) cancer staging confirmed 6 patients with stage I disease, 13 with stage II and 18 patients with stage III disease (**Table 2**). Seven patients received neo-adjuvant therapy and eighteen patients received adjuvant chemotherapy after surgery. Two patients in the neo-adjuvant group received concurrent chemo-radiation and the majority of patients received platinum/fluorouracil based combination chemotherapy (**Table 3**). At a mean follow-up of 12.06 months, thirteen patients developed recurrence, 12 patients with distant recurrence and 1 patient for which data was not reported. Of the 12 patients with distant recurrence, 3 also had local recurrence. Ten patients (77%) had died at the time of long-term follow-up.

**Table 1:** Characteristics of the 37 patients included in the study.

Patients	N = 37
Age	62.7 (range, 44–90)
Sex, M∶F	30∶7
Stage	Stage I, 6 (16.2%)
	Stage II, 13 (35.2%)
	Stage III, 18 (48.6%)
Peri-operative therapy	Neoadjuvant therapy, 7 (19%)
	Adjuvant chemotherapy, 18 (48.6%)
	No peri-operative therapy, 12 (32.4%)
Recurrence (N = 13)	Stage I, 0 (0%)
	Stage II, 3 (23%)
	Stage III, 10 (77%)
Follow-up period (month)	12 months (range, 0.4–30.3)

**Table 2:** Stage of Tumor by Survivin Expression Level.

	Adjacent Survivin Epithelium Expression		Tumor Survivin Expression	
	Low Risk	High Risk	Low expression	High expression
Stage I	5	1	0	6
Stage IIa	2	2	1	3
Stage IIb	6	3	4	5
Stage III	10	8	5	13
Total	37		37	

**Table 3:** Chemotherapy by Survivin Expression Level.

	Adjacent Survivin Epithelium Expression		Tumor Survivin Expression	
	Low Risk	High Risk	Low expression	High expression
Neo-Adjuvant	0	7*	0	7
Adjuvant	12	6	8	10
No Treatment	11	1	2	10
Total	37		37	

*2 patients received concurrent chemoradiation.

**Figure 5:**
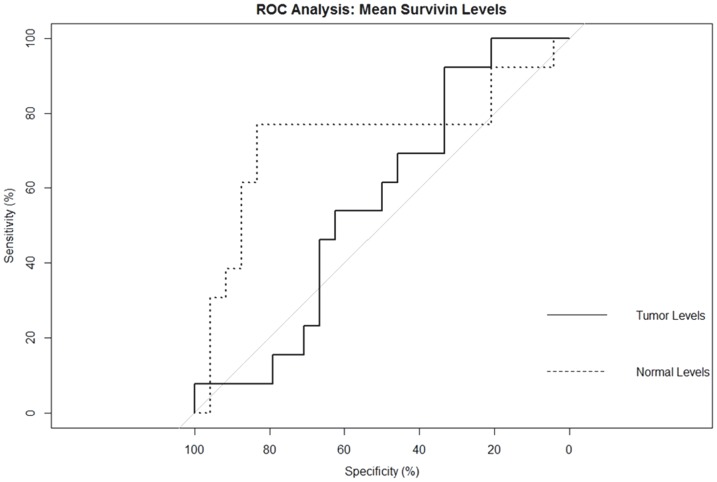
ROC analysis to correlate survivin levels and recurrence. The probability for distant recurrence was determined for survivin levels in human EAC tumor and adjacent squamous epithelial tissue. An increased survivin level in adjacent squamous epithelial tissue was determined to be a risk factor for distant recurrence (p = 0.02).

ROC analysis indicated that a threshold of greater than or equal to 2.85 for mean survivin mRNA expression in CASE was indicative of a heightened risk for distant recurrence with a sensitivity of 0.77, specificity of 0.83, and an AUC of 0.73 (p<.05. PPV = 0.71, NPV = 0.87) (**Figure 5**). Thus patients exhibiting mean survivin expression of 2.85 or greater in CASE were classified into the high-risk group and those with expression levels of less than 2.85 were categorized into the low risk group. Despite a normal histologic appearance, survivin overexpression in CASE proved to be an indicator of distant recurrence. Survivin expression in tumor tissue did not correlate with recurrence. Categorization into high and low risk groups using a threshold *tumor* survivin expression of 3.66 did not predict recurrence (p = 0.55) but did predict an increase in the odds of death from EAC (described subsequently).

**Figure 6:**
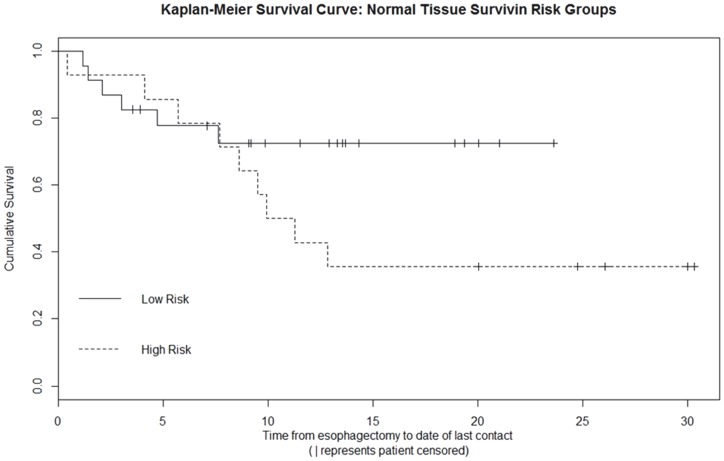
Kaplan-Meier survival curve based on high and low risk groups. Kaplan-Meier survival curves were generated for high and low risk groups based on adjacent squamous epithelial survivin CASE expression. The high-risk group demonstrated an association with increased mortality, although the correlation did not reach significance. Kaplan-Meier analysis for tumor survivin high and low risk groups yielded similar results (p = 0.11).

Kaplan-Meier survival curves were generated for high and low risk groups based on CASE survivin expression. The high-risk group demonstrated an association with increased mortality, although the correlation did not reach significance (p = 0.12, **Figure 6**). Kaplan-Meier analysis for tumor survivin high and low risk groups yielded similar results (p = 0.11).

A Cox proportional hazards model was used to assess the effectiveness of survivin level within both CASE and EAC tumor tissue samples in predicting mortality. Other risk factors considered were age at time of esophagectomy, tobacco use (in packs per years), alcohol consumption, and a history of Barrett's esophagus (**Table 4**). Smoking was associated with a higher death rate (p = 0.06) with the odds of mortality being 1.018 greater for each pack per year smoked. Survivin expression in tumor tissue was associated with increased risk of death (p = 0.05). The probability of a patient in the high-risk tumor survivin group experiencing death was 5.23 times greater than that of a patient categorized in the low risk group (p<.05).

**Table 4:** Cox Regression Survival Analysis.

Variable	Omnibus Statistic	X^2^ (Wald)	*p*-value	Odds Ratio	Odds Ratio 95% Confidence Interval
Block	G^2^ (6, N = 37) = 10.82		0.094		
Alcohol		1.86	0.172	0.692	0.416–1.17
Smoking^1^		3.58	0.058	1.018	0.999–1.04
Age^2^		0.42	0.515	1.019	0.963–1.08
Barrett's^3^		2.97	0.085	0.293	0.072–1.19
Risk: S^4^		2.28	0.131	2.55	0.758–8.55
Risk: T^5^		3.88	0.049	5.23	1.01–27.03

1 In pack year.

2 At time of esophagectomy.

3 History of Barrett's Esophagus.

4 High risk group determined by adjacent squamous epithelial tissue mean Survivin levels.

5 High risk group determined by tumor tissue mean Survivin levels.

## Discussion

Studies have shown higher levels of survivin in metaplastic columnar epithelium and dysplastic Barrett's epithelium compared to squamous tissue thus supporting the hypothesis that upregulation of this gene is likely an early event preceding development of adenocarcinoma [12]. Reports have also supported survivin as a potential biomarker in esophageal squamous cell carcinoma [28] but there is conflicting evidence regarding its prognostic role in esophageal adenocarcinoma [29]. In a study by Rosato and colleagues, survivin expression had no prognostic value for patients with EAC. [9] The aim of the current study was to evaluate the role of this anti-apoptotic gene in greater depth in esophageal adenocarcinoma and CASE taking into consideration tumor heterogeneity as well as expression of genetic changes prior to histologic manifestation of tumor phenotype in adjacent epithelium.

Analysis of human tissue using IHC, Western blot, and qRT-PCR confirmed upregulation of survivin in EAC tumor tissue compared to CASE. Our results established that increased survivin expression in EAC tumor tissue is a risk factor for death with the high-risk tumor survivin group being 5 fold more likely to die than those categorized in the low-risk survivin group. It is possible that the higher expression within the EAC tumor tissue may relate to the aggressiveness of the tumor (i.e., worsening cellular dysregulation), hence correlating with mortality. However, based on our sample size, this study is likely underpowered and larger series of patients would be needed to study these associations further.

Additionally expression of survivin in CASE was not altogether absent, as would be expected for terminally differentiated normal squamous epithelium [10], [11]. Considering evidence demonstrating upregulation of this gene in pre-malignant metaplastic and dysplastic columnar epithelium, our data suggest that survivin overexpression may be an early genetic event occurring in histologically normal appearing squamous epithelium prior to development of metaplasia and transformation to adenocarcinoma. Though the mucosa adjacent to the tumor maintains squamous differentiation, it harbors overexpression of this anti-apoptotic gene that could be a potential driver of tumorigenesis. Alternatively, it could be postulated that based on the correlation with recurrence, survivin expression in CASE could be thought of as the molecular equivalent of a positive margin and/or a predictor of distant recurrence. If elevated survivin levels in CASE represents molecular evidence that adjacent tissue is showing signs of malignancy, the prognosis may be worse than that predicted by the pTNM classification system. It could be postulated that the fact that there was not an increase in mortality in patients with elevated survivin levels in CASE could represents a type II error secondary to the small sample size. Larger studies may help clarify these associations.

The fact that survivin expression in tumor did not correlate with recurrence could be from the fact that these patients had died before recurrence could have been documented; this supposition is supported by the fact that there were more patients with advanced stage disease in the high-risk tumor expression group. Moreover, as evidenced from prior studies, each tumor exhibits significant heterogeneity with intra-tumor variations in somatic mutations, allelic composition, tumor suppressor and oncogenic dysregulation [30]. The predominance of multiple unique clones in sampled tumor tissue may lead to inconsistent results when performing molecular profiling of a given cancer. In the present study, fold change in survivin mRNA was highly variable in tumor tissue in comparison to CASE, and this may reflect tumor heterogeneity and provide a potential explanation for the lack of correlation with tumor recurrence. CASE demonstrated more consistent levels and is perhaps a better substrate for evaluation of survivin as a prognostic marker in patients with EAC. These data underscore the need for a concerted effort to bank and analyze cancer-adjacent histologically normal appearing tissue for molecular profiling in EAC and perhaps other tumors.

Cell line analysis in the present study confirmed clear inhibition of survivin through transfection with siRNA. Survivin was effectively downregulated and cleaved caspase 3 was consumed in the reaction leading to upregulation of cleaved PARP, indicating that inhibition of survivin leads to activation of the apoptotic pathway. As survivin is not expressed in normal tissue, it is potentially an ideal target for novel agents [10], [11]. Therefore, effective inhibition through siRNA with downstream activation of apoptosis supports the validity of future clinical trials in EAC evaluating the effectiveness of a novel agent that uses siRNA or other inhibitory avenues [10].

In conclusion the present study supports upregulation of survivin as a potential early genetic change in EAC occurring in normal appearing CASE. Survivin expression within EAC tissue was a significant predictor of mortality while the expression level of survivin in CASE was a more reliable predictor of tumor recurrence. Additionally we also demonstrated that inhibition of survivin in EAC cell lines leads to upregulation of apoptosis supporting further evaluation of therapeutic strategies targeted to this marker.

The limitations of the study include the small sample size and a relatively shorter duration of follow up. Further studies with larger sample sizes and longer follow up may help clarify some of the associations noted in studies on survivin expression in EAC and CASE. This would also help control for more factors impacting recurrence and survival in patients with EAC.
